# DNA Damage Response and Redox Status in the Resistance of Multiple Myeloma Cells to Genotoxic Treatment

**DOI:** 10.3390/ijms262010171

**Published:** 2025-10-19

**Authors:** Panagiotis Malamos, Christina Papanikolaou, Elisavet Deligianni, Dimitra Mavroeidi, Konstantinos Koutoulogenis, Maria Gavriatopoulou, Evangelos Terpos, Vassilis L. Souliotis

**Affiliations:** 1 Institute of Chemical Biology, National Hellenic Research Foundation, 116 35 Athens, Greece; pmalamos@eie.gr (P.M.); chrpapa@eie.gr (C.P.); edelig@eie.gr (E.D.); dmavro@eie.gr (D.M.); 2 Department of Nutrition and Dietetics, School of Health Science and Education, Harokopio University, 176 76 Athens, Greece; konkout@hua.gr; 3 Department of Clinical Therapeutics, School of Medicine, National and Kapodistrian University of Athens, 115 28 Athens, Greece; mgavria@med.uoa.gr (M.G.); eterpos@med.uoa.gr (E.T.)

**Keywords:** multiple myeloma (MM), DNA damage response (DDR), oxidative stress, chromatin condensation, resistance to genotoxic treatment, nucleotide excision repair (NER), apurinic/apyrimidinic sites, interstrand crosslinks (ICL), apoptosis rates, melphalan

## Abstract

The DNA Damage Response (DDR) network is an essential machinery for maintaining genomic integrity, with DDR defects being implicated in cancer initiation, progression, and treatment resistance. Moreover, oxidative stress, an imbalance between reactive oxygen species production and antioxidant defense, can significantly impact cell viability, leading to cell death or survival. Herein, we tested the hypothesis that DDR-related signals and redox status measured in multiple myeloma (MM) cell lines correlate with the sensitivity to genotoxic insults. At baseline and following irradiation with Ultraviolet C (UVC; 50 J/m^2^) or treatment with melphalan (100 μg/mL for 5 min) DDR-related parameters, redox status expressed as GSH/GSSG ratio and apurinic/apyrimidinic sites were evaluated in a panel of eleven human MM cell lines and one healthy B lymphoblastoid cell line. We found that MM cell lines with increased apoptosis rates displayed significantly higher levels of endogenous/baseline DNA damage, reduced GSH/GSSG ratio, augmented apurinic/apyrimidinic lesions, decreased nucleotide excision repair and interstrand crosslinks repair capacities, and highly condensed chromatin structure. Taken together, these findings demonstrate that DDR-related parameters and redox status correlate with the sensitivity of MM cells to DNA-damaging agents, specifically melphalan, and, if further validated, may be exploited as novel sensitive/effective biomarkers.

## 1. Introduction

Multiple myeloma (MM) is a hematological malignancy characterized by the clonal expansion of malignant plasma cells. The disease is usually preceded by asymptomatic precursor conditions, namely monoclonal gammopathy of undetermined significance (MGUS) and smoldering multiple myeloma (sMM) [1]. MGUS has an annual risk of progression ranging at 1%, while sMM represents an intermediate state with increased progression rates up to 10% per year for the first five years after diagnosis [2,3]. MM accounts for 10% of all hematologic malignancies and is slightly more common in men, with age ranging at 66–70 years at diagnosis [4,5,6]. In many patients, genetic heterogeneity, chromosomal instability, and replication stress are present. These events accumulate during the transformation process of myelomagenesis and progressively complicate the disease landscape [7,8]. Today, treatment approaches vary based on disease stage, patient health, and individual needs, often involving a combination of therapies. Key treatments include chemotherapy, targeted therapy, immunotherapy, and stem cell transplant [9]. A common starting point in ΜΜ treatment involves chemotherapy, with the primary goal of reducing the number of abnormal plasma cells. Melphalan-based treatments have long served as a gold-standard therapy, exploiting the vulnerability of cancer cells to DNA damage. However, treatment often fails due to pre-existing and therapy-induced resistance, as cancer cells escape its effects [10,11,12]. Drugs like proteasome inhibitors (bortezomib, carfilzomib, ixazomib), immunomodulatory drugs (ImiDs; thalidomide, lenalidomide, pomalidomide), and B-cell maturation antigen (BCMA)-targeted therapies are also used to target specific proteins on myeloma cells [13,14,15]. Interestingly, Chimeric antigen receptor (CAR) T-cell therapy, a type of immunotherapy that modifies a patient’s own T-cells to target and destroy cancer cells, has shown promise in treating MM, particularly in relapsed or refractory cases [16]. Autologous stem cell transplant (using the patient’s own cells) is often used, especially for eligible patients, to replace damaged bone marrow cells with healthy ones [17].

MM is characterized by increased oxidative stress, partly due to the augmented production of immunoglobulins by plasma cells, which leads to endoplasmic reticulum stress and the generation of reactive oxygen species (ROS) [18]. A growing number of studies have shown that oxidative stress contributes to the development and progression of MM, as well as influencing the response to treatment and the development of drug resistance [19,20]. Indeed, the redox status of MM cells plays a crucial role in their response to therapies like proteasome inhibitors, with recent studies showing that the increase in oxidative stress can be a strategy to induce cell death in MM cells [19]. On the other hand, MM cells adapt to the increased oxidative stress by developing antioxidant defense mechanisms. This adaptation can lead to chemoresistance, as the cells become more capable of neutralizing the effects of drugs that induce oxidative stress [21]. The intimate relationship between redox status, MM progression, and treatment response has made it a target for potential therapeutic interventions. Oxidative stress can be measured directly by detecting ROS or indirectly by assessing the damage they cause to biomolecules, such as lipids, proteins, and nucleic acids. Several markers are used to assess oxidative stress in MM, including malondialdehyde and advanced oxidation protein products, which are found at higher levels in MM patients compared to healthy individuals [22]. MM cells also exhibit altered levels of glutathione, a key antioxidant tripeptide, as well as antioxidant enzymes, like superoxide dismutase, glutathione peroxidase, and catalase [19]. The ratio of reduced (GSH) to oxidized (GSSG) glutathione can serve as a critical indicator of cellular redox status.

The genome of all organisms is under continuous threat by both endogenous factors, such as oxidation, DNA alkylation, hydrolysis, mismatching of DNA bases, and replication fork collapse, as well as by external insults, like chemotherapeutic agents and radiation, including ultraviolet (UV) and ionizing radiation. To maintain their genomic integrity, cells have evolved a complex machinery of sensors and effectors known as the DNA Damage Response (DDR) network [23]. This network can detect DNA lesions and orchestrate their repair through the coordinated action of DDR pathways. Several deficiencies in core molecules of DDR influence the cells’ fate for the detection and repair of DNA lesions and lead to genomic instability, which is a hallmark of cancer [24]. In MM, an increasing body of evidence highlights the critical role that DDR aberrations play in the pathogenesis and progression of the disease as well as in the resistance to therapy [12,25,26]. These aberrations might be the result of a fundamental defect in the malignant plasma cells’ capacity to recognize and remove errors, leading to a mutator phenotype [27]. A variety of genetic alterations and abnormalities are acquired by malignant plasma cells throughout the onset and progression of MM. In fact, loss-of-function mutations in critical DDR components, such as ATM, TP53, and TP73, are common in MM and hinder the apoptotic response to DNA damage [12]. Interestingly, more severe disease and treatment resistances are correlated with the activation of DNA repair mechanisms to correct these DNA errors [12].

In this study, we tested the hypothesis that DDR-associated parameters and redox status correlate with the sensitivity to genotoxic insults. Therefore, in a panel of human myeloma cell lines we measured apoptosis rates, endogenous/baseline DNA damage, critical DNA repair mechanisms, namely nucleotide excision repair (NER), interstrand crosslinks (ICLs) repair and double-strand breaks (DSBs) repair, the degree of chromatin condensation, the redox status, and apurinic/apyrimidinic lesions. Our findings revealed a substantial heterogeneity in redox status and DDR function across the MM cell lines analyzed, identifying specific subgroups that may be more vulnerable to genotoxic treatment, specifically melphalan.

## 2. Results

### 2.1. MM Cell Lines Showed Differential Sensitivity to Genotoxic Insults

**Figure 1:**
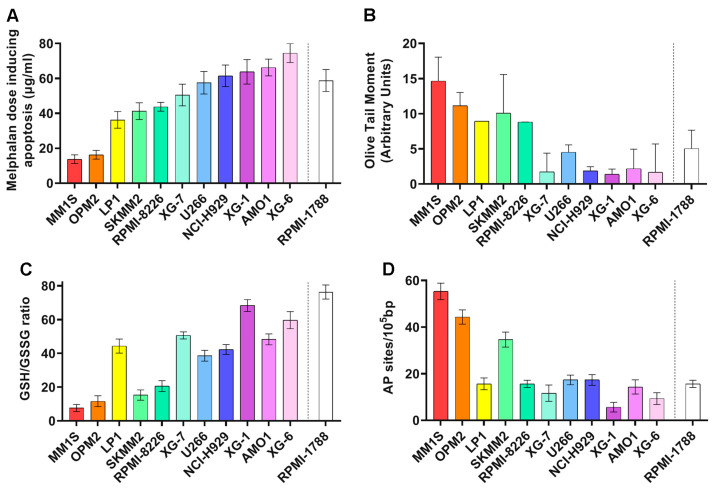
MM cell lines’ sensitivity to genotoxic insults and DDR-related parameters at baseline. (**A**) Bar charts showing the apoptosis rates 24 h following treatment with 0–100 μg/mL melphalan for 5 min. (**B**) Baseline DNA damage assessed by alkaline comet assay. (**C**) GSH/GSSG ratio at baseline. (**D**) Endogenous AP-sites. The data represent the mean of at least three independent experiments. Error bars indicate standard deviation (SD). The dashed line denotes the healthy control (RPMI-1788).

Firstly, in eleven human MM cell lines (HMCLs) and one healthy B lymphoblastoid cell line the apoptosis rates as a marker of cells’ sensitivity to genotoxic insults were measured. To trigger apoptosis, cell lines were treated in vitro with increasing doses (0–100 μg/mL) of melphalan for 5 min and apoptosis rates were measured 24 h after treatment. We found that HMCLs exhibited varying rates of apoptosis (Figure 1A). Interestingly, the highest doses of melphalan required to trigger apoptosis were observed in AMO1 and XG-6 cell lines, indicating that these cell lines are characterized by the lowest apoptosis rates. On the other hand, MM1S and OPM2 showed the highest apoptosis rates.

### 2.2. DDR-Related Parameters in MM Cell Lines at Baseline

To investigate the basis of this differential sensitivity to genotoxic insults, we evaluated several DDR-related factors at baseline. The presence of the endogenous/baseline DNA damage was measured using comet assay under alkaline conditions (Figure 1B). HMCLs showed differential DNA damage burden, with MM1S and OPM2 showing the highest values while AMO1 and XG-6 showed the lowest ones. Moreover, we assessed crucial factors, namely redox dysregulation and apurinic/apyrimidinic sites that contribute to the intracellular production of SSBs and DSBs. MM cell lines also showed differential levels of these factors examined. Particularly, MM1S and OPM2 cell lines showed the lowest GSH/GSSG ratio (Figure 1C) and the highest apurinic/apyrimidinic sites (Figure 1D). Moreover, AMO1 and XG-6 cells exhibited relatively high GSH/GSSG ratio and reduced apurinic/apyrimidinic sites.

### 2.3. NER Capacity of MM Cell Lines

**Figure 2:**
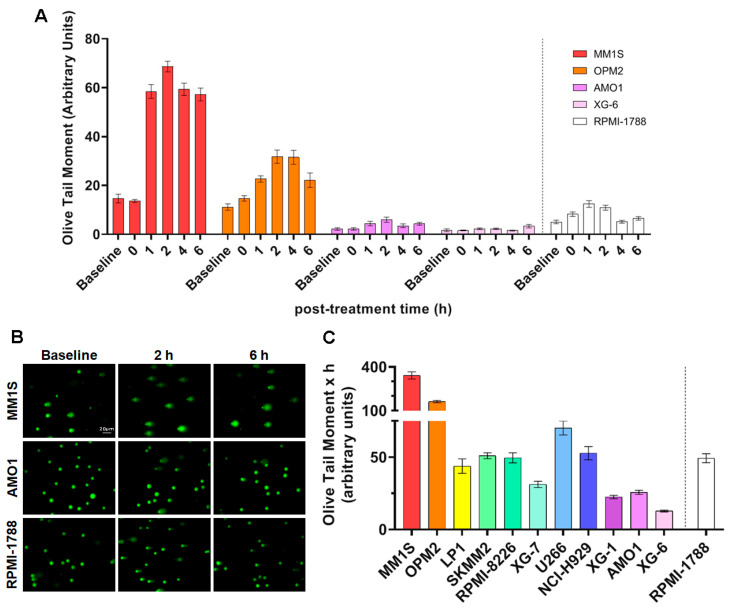
UVC-induced DDR signals in MM cell lines. (**A**) Bar graphs of the kinetics of UVC-induced NER using alkaline comet assay for selected samples. (**B**) Representative images from alkaline comet assay of MM1S, AMO1, and RPMI-1788 (healthy) cells at baseline and at 2 h and 6 h after UVC irradiation (50 J/m^2^), scale bar: 20 μm. (**C**) Cumulative DNA damage levels quantified as AUC over the entire 0–6 h time course in all samples. A minimum of three biological independent experiments were performed for each condition. Error bars represent SD. The dashed line denotes the healthy control (RPMI-1788).

For NER studies, HMCLs were irradiated by increasing Ultraviolet C (UVC) doses (0–100 J/m^2^), which led to the formation of 6-4 photoproducts (6-4PPs) and cyclobutane pyrimidine dimers (CPDs), i.e., DNA adducts that are repaired almost exclusively by the NER mechanism. Viability assay [3-(4,5-dimethylthiazol-2-yl)-2,5 diphenyl tetrazolium bromide; MTT] showed the appropriate UVC dose, with a viability threshold of greater than 70% (Figure S1). The optimum dose in this experimental procedure was adjusted at 50 J/m^2^ and alkaline comet assay was then performed upon the desired timepoint of 0, 1, 2, 4, and 6 h. In all cell lines, maximal levels of DNA damage were observed at 2 h following UVC irradiation, decreasing thereafter (Figure 2A,B and Figure S2A). Significant differences in the efficiencies of NER [expressed as the Area Under the Curve (AUC) for DNA adducts during the experiment (0–6 h)] were observed between the HMCLs analyzed (Figure 2C). Interestingly, MM1S and OPM2 that are characterized by the highest levels of apoptosis, endogenous/baseline DNA damage and AP-sites, as well as by the lowest GSH/GSSG ratio, also showed the highest AUC values, indicating the worst capacity for NER. On the other hand, AMO1 and XG-6 (cell lines with the lowest levels of apoptosis, endogenous/baseline DNA damage, and AP-sites, as well as the highest GSH/GSSG ratio) showed the best NER capacity.

**Figure 3:**
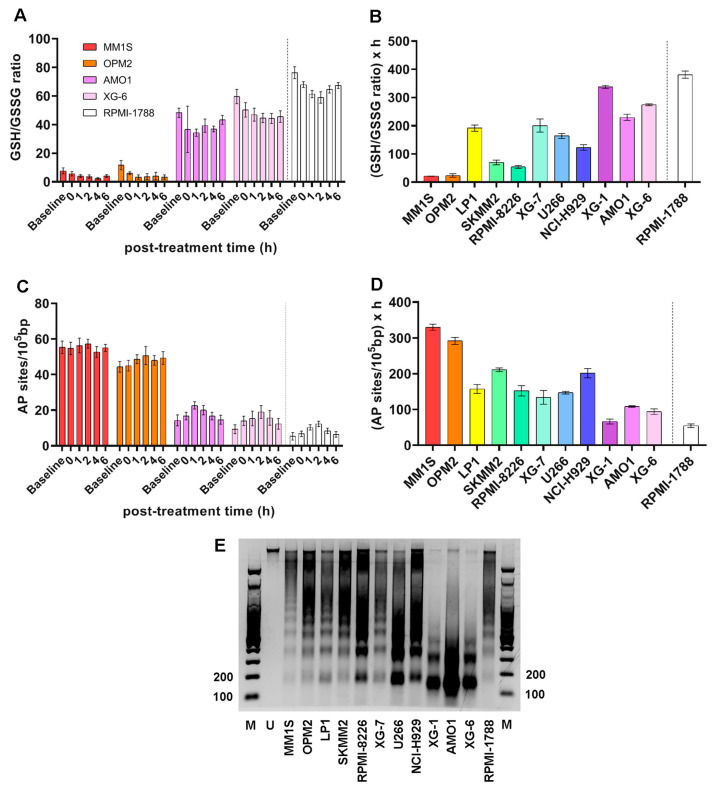
UVC-induced changes in the redox state and chromatin condensation of MM cell lines at baseline. (**A**) GSH/GSSG ratio in representative cells after UVC (50 J/m^2^) and (**B**) total GSH/GSSG ratio as AUC across all samples. (**C**) AP-sites formation in representative cells after UVC irradiation and (**D**) total amounts of AP-sites quantified as AUC. A minimum of three biological independent experiments were performed for each condition. Error bars represent SD. (**E**) Representative autoradiograms showing chromatin condensation of the MM and healthy cell lines in the *N-ras* gene. M, 100 bp DNA Ladder marker. U, untreated sample. The dashed line denotes the healthy control (RPMI-1788).

Furthermore, UVC-induced decrease in GSH/GSSG ratio (Figure 3A,B and Figure S2B) and increase in AP-sites (Figure 3C,D and Figure S2C) were observed in all treated samples, with MM1S and OPM2 showing the lowest GSH/GSSG ratio and the highest AP-sites levels, while on the contrary AMO1 and XG-6 showed the highest GSH/GSSG ratio and the lowest AP-sites.

Previous studies have shown that nucleotide excision repair is greatly affected by the local chromatin structure [28], and that the NER efficiency of the *N-ras* gene correlates with melphalan-induced apoptosis [29,30]. Therefore, herein, we analyzed chromatin condensation in the *N-ras* gene, using micrococcal nuclease digestion of HMCLs at baseline. As shown in Figure 3E, MM cell lines showed varying degrees of chromosomal condensation. That is, AMO1 and XG-6 cell lines, which are characterized by high DNA repair capacities, showed greater looseness of chromatin structure, since the *N-ras* gene gave rise mostly to mono- and di-nucleosome structures, with a significant portion in mono-nucleosomes. On the other hand, the same gene in cell lines that showed low DNA repair capacity (MM1S and OPM2) showed condensed chromatin, since it gave rise to di- and tri-nucleosomes and higher structures.

### 2.4. ICL Formation and Repair Capabilities in MM Cell Lines

**Figure 4:**
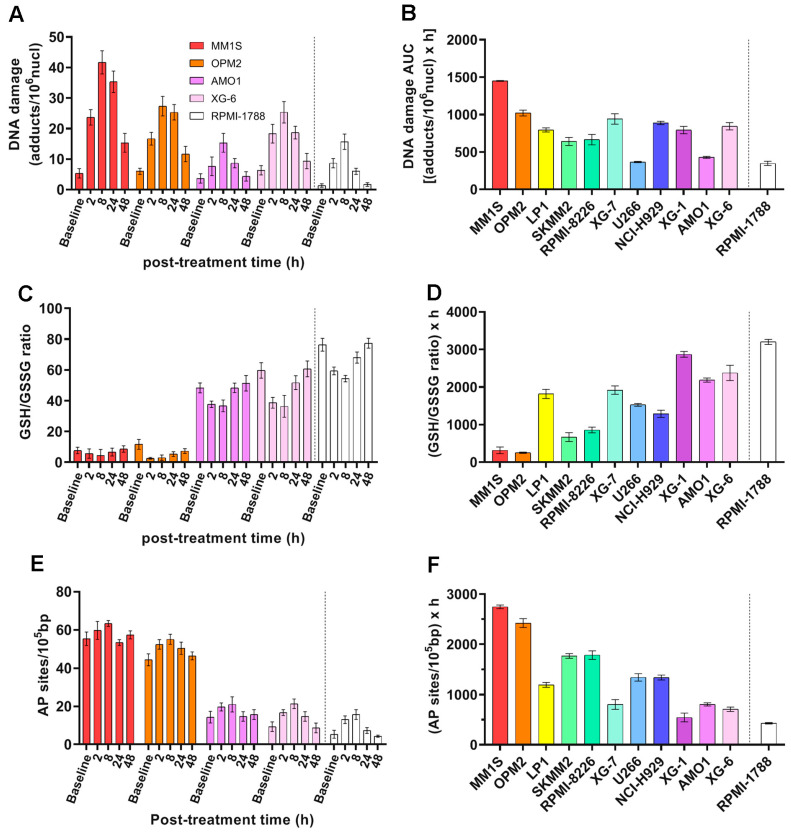
DDR-related signals in MM cells following melphalan treatment. (**A**) Bar graphs of the kinetics of ICLs formation and repair in the *N-ras* gene upon treatment of representative cells with 100 μg/mL melphalan for 5 min. (**B**) Total ICL repair (ICL/R) capacity quantified as AUC. (**C**) GSH/GSSG ratio kinetics in selected samples following melphalan exposure and (**D**) total GSH/GSSG ratio across all samples quantified as AUC. (**E**) Selected cell’s AP-sites formation post-treatment and (**F**) total amounts of AP-sites measured as AUC. All experiments were conducted in at least three independent replicates. Error bars indicate SD. The dashed line denotes the healthy control (RPMI-1788).

To measure the efficiency of the ICL repair, HMCLs were treated with 100 μg/mL melphalan for 5 min, and the ICL formation/repair in the *N-ras* gene was followed for 0, 2, 8, 24, and 48 h after treatment (Figure 4A,B and Figure S3A). In all cell lines examined, maximal ICL levels were obtained at 8 h following melphalan treatment. In addition, a melphalan-induced decrease in the GSH/GSSG ratio (Figure 4C,D and Figure S3B) and increase in AP-sites (Figure 4E,F and Figure S3C) were found in all HMCLs analyzed. In line with the NER results, following melphalan treatment MM1S and OPM2 showed the worst ICL repair capacity, the lowest GSH/GSSG ratio, and the highest AP-sites.

### 2.5. DSB Repair Capacity of MM Cell Lines

**Figure 5:**
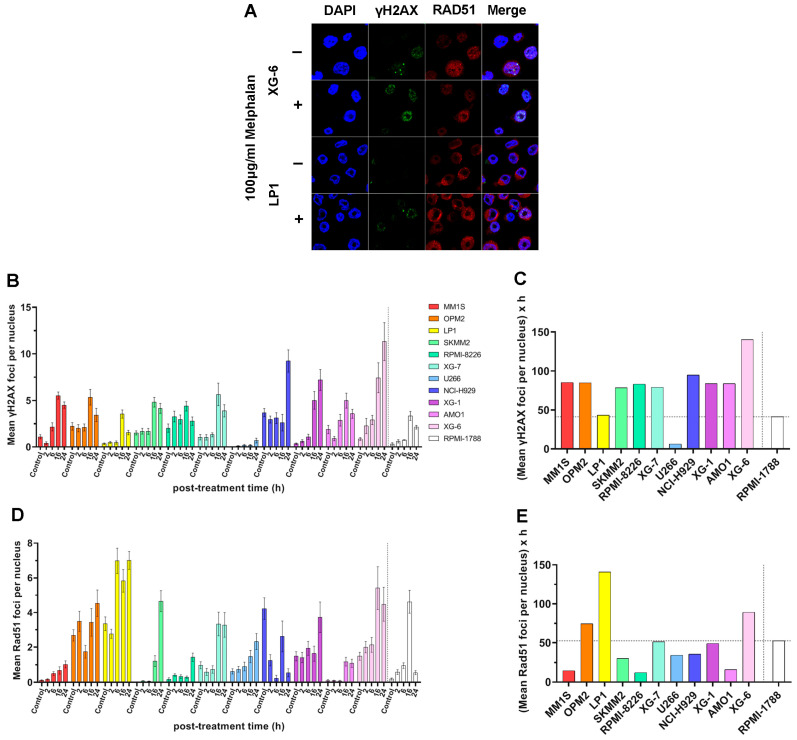
DSB repair capacity of MM cell lines in response to treatment. (**A**) Representative confocal images of XG-6 and LP1 cells at baseline (−, untreated) and at 16 h following treatment with 100 μg/mL melphalan for 5 min (+, treated). Nuclei are stained with DAPI (blue), γH2AX foci (green) and RAD51 (red). Magnification ×630. (**B**) Bar graphs showing the kinetics of mean γH2AX foci per nucleus following melphalan treatment. (**C**) Total γH2AX foci levels quantified as AUC post-treatment. (**D**) Bars present the kinetics of mean RAD51 foci per nucleus after stimulation with melphalan. (**E**) Total RAD51 foci levels expressed as AUC. Each experiment was repeated independently at least three times. Error bars represent SD. The dashed line denotes the healthy control (RPMI-1788).

To evaluate DSB repair efficiency, we monitored γH2AX and RAD51 foci kinetics across HMCLs following treatment with 100 μg/mL melphalan for 5 min (Figure 5A). Formation of γH2AX foci was rapidly induced in all cell lines, with peak levels typically observed between 16 and 24 h post-treatment (Figure 5B). However, the pattern of γH2AX accumulation in the cell lines examined was different from that found in the other DDR-associated parameters mentioned above. Indeed, XG-6 cell line showed maximal levels of γH2AX accumulation with the MM1S, OPM2, SKMM2, RPMI-8226, XG-7, XG-1, and AMO1 exhibiting similar AUC values (Figure 5A–C). In parallel, HMCLs showed differential RAD51 foci kinetics, with LP1, OPM2, and XG-6 displaying robust RAD51 induction and high RAD51 AUC values, whereas MM1S, SKMM2, RPMI-8226, U266, and AMO1 showed significantly lower RAD51 responses (Figure 5D,E).

### 2.6. Statistical Analysis of DDR Parameters Across MM Cell Lines

**Figure 6:**
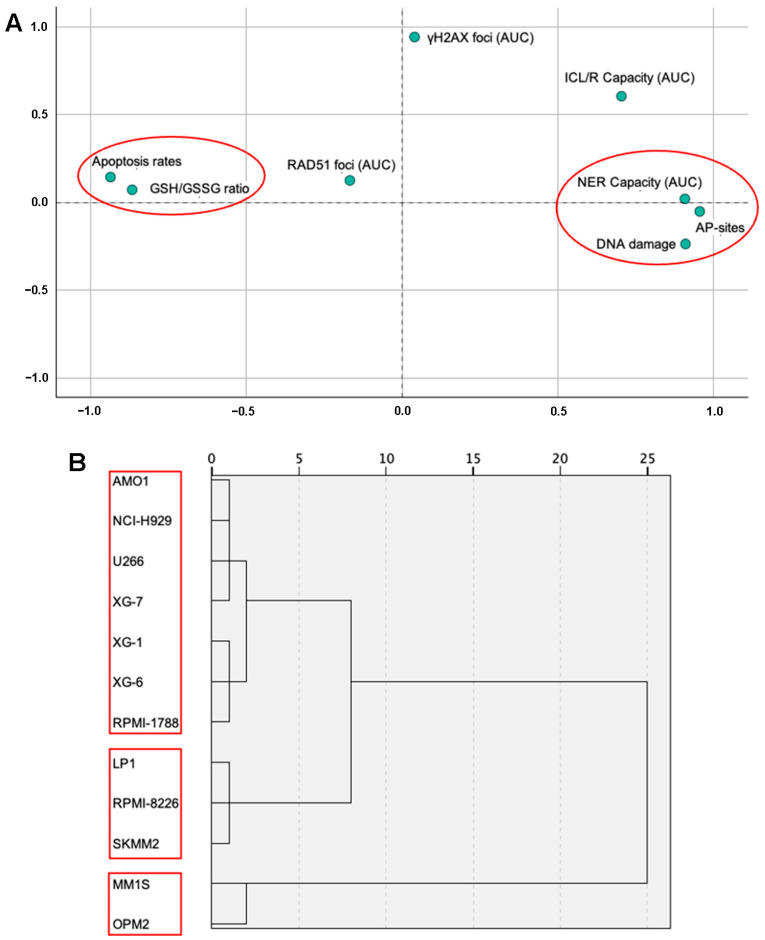
Statistical analysis of DDR markers in MM cell lines. (**A**) Principal component analysis (PCA) reveals two distinct factors (red circles) underlying the distribution of the markers across the cell lines. (**B**) Hierarchical clustering of Z-score normalized DDR marker values, using Ward’s method, identifies three distinct clusters (red boxes) among the samples.

In the same eleven HMCLs and one healthy B lymphoblastoid cell line, eight molecular markers were analyzed (apoptosis rates, endogenous/baseline DNA damage levels, GSH/GSSG ratio, and AP-sites as well as repair mechanisms; NER, ICL/R, and DSB repair involvement with foci, such as γH2AX and RAD51). The results of the correlation analysis between the markers of the cell lines are presented in Table 1. Apoptosis rates were negatively correlated with all the markers (DNA damage: Rho = −0.918, *p* = 0.01; AP-sites: Rho = −0.864, *p* = 0.01; NER capacity: Rho = −0.792, *p* = 0.01; ICL/R capacity: Rho = −0.617, *p* = 0.05) except GSH/GSSG ratio (Rho = 0.792, *p* = 0.01) which was positively correlated. DNA damage was negatively correlated with GSH/GSSG ratio (Rho = −0.804, *p* = 0.01) and positively with AP-sites (Rho = 0.864, *p* = 0.01) and NER capacity (Rho = 0.762, *p* = 0.01). Finally, AP-sites were positively correlated with NER capacity (Rho = 0.892, *p* = 0.01) and ICL/R capacity (Rho = 0.592, *p* = 0.05), as well as NER capacity with ICL/R capacity (Rho = 0.707, *p* = 0.05). No correlation was found between foci and the rest of the markers. PCA is illustrated in Figure 6A. The KMO test was calculated at 0.546 and Bartlett’s test of sphericity was <0.001. The analysis revealed a distinct pattern of two factors. The first factor consisted of Apoptosis rate and GSH/GSSG ratio, while the second of NER capacity, AP-sites, and DNA Damage. Additionally, a dendrogram is depicted in Figure 6B representing a further hierarchical analysis of the markers in terms of the cell lines. For this analysis, foci and ICL/R capacity markers were excluded as no statistical significance was shown after Pearson correlation or factor analysis through PCA. Hierarchical analysis comprised a pattern of three clusters solution for the cell lines. Firstly, Group A, consisting of MM1S and OPM2, the second of LP1, RPMI-8226, and SKMM2 as Group B, followed by Group C, including RPMI-1788, XG-6, XG-1, XG-7, U266, NCI-H929, and AMO1.

After grouping of the cell lines as hierarchical clustering determined, ANOVA statistical analysis revealed significant differences among the three groups and the measured molecular markers (Table S1 and Figure S4). Apoptosis rates were lower in ascending order when comparing Group A vs. B (15 vs. 40.41667, *p* = 0.006) and Group A vs. C (15 vs. 61.82143, *p* < 0.001), as well as Group B vs. C (40.41667 vs. 61.82143, *p* = 0.003). As for endogenous/baseline DNA damage, the results showed that Group C had higher in vitro implications when compared to both Group A (2.63062 vs. 12.8952, *p* < 0.001) and Group C (2.63062 vs. 9.26916, *p* < 0.001). GSH/GSSG ratio was significantly lower in Group A vs. C (9.66667 vs. 54.90476, *p* = 0.007). AP-sites demonstrated a significant difference pattern between Group A and C (49.83333 vs. 13.04762, *p* < 0.001). NER and ICL/R capacity appeared borderline statistically different between Group A and C (252.045 vs. 37.75, *p* = 0.048) as well as Group A and B (12345.5 vs. 700.11111, *p* = 0.047). Finally, no significant differences were observed in the γH2AX and RAD51 foci molecular markers among the groups.

## 3. Discussion

DDR is a network of molecular pathways that is responsible for the removal of DNA damage and maintenance of genomic stability. Dysregulation of this system plays a central role in oncogenesis and therapy outcome in cancer. Herein, to understand the molecular basis of the link between DNA damage formation/repair and the response to genotoxic therapy, we assessed critical DDR-related signals, chromatin condensation, and redox status in a panel of HMCLs with different sensitivity to DNA-damaging insults, specifically melphalan.

Firstly, cell lines were classified according to their sensitivity to genotoxic agents, with apoptosis being used as a marker of a cell’s sensitivity. Indeed, apoptosis can indicate the cell’s responsiveness to different stimuli, especially regarding disease or therapy [31]. The degree to which cells experience apoptosis when exposed to a specific trigger can reveal their susceptibility or resilience to this factor. In this study, to trigger apoptosis, cell lines were treated with melphalan, a bifunctional alkylating agent that binds to DNA and forms several DNA lesions, including monoadducts and ICLs [32]. Prior research has shown that monoadducts are repaired almost exclusively by NER, while ICLs are repaired by a complex process that requires the involvement of NER, the Fanconi anemia pathway, translesion synthesis, homologous recombination (HR), and nonhomologous end-joining (NHEJ) [33].

Variations in the apoptosis rates between different HMCLs were observed. To understand the origin of these variations, we investigated DDR-associated parameters and redox status that play significant roles in determining the cell’s fate, influencing whether the cell repairs the lesion and survives, or undergoes apoptosis if the damage is extensive or cannot be fixed. We found that HMCLs with increased apoptosis rates are characterized by higher levels of endogenous/baseline DNA damage, reduced NER and ICL repair capacities, and condensed chromatin structure, as well as by reduced GSH/GSSG ratio and augmented apurinic/apyrimidinic lesions.

Differential endogenous/baseline DNA damage burden was found in the HMCLs analyzed, with MM1S and OPM2 exhibiting the highest and AMO1 and XG-6 the lowest levels. Endogenous/baseline DNA damage is a danger to cellular health and survival, since it can cause mutations, genomic instability, and cell death, including apoptosis. In line with our data, a previous study demonstrated increased levels of damage in several HMCLs, apart from U266 [34]. Moreover, the authors reported that HMCLs with persistent endogenous DNA damage rely on HR over NHEJ and ATR over ATM, indicating the presence of replication stress in these cells. Another study demonstrated increased endogenous DNA damage in HMCLs, as well as in peripheral blood mononuclear cells (PBMCs) and bone marrow plasma cells (BMPCs) from patients with MGUS, sMM, and MM, suggesting that this ongoing DNA damage is not merely a consequence of myeloma but also is involved in the progression and development of the disease [35]. In a different investigation, the authors reported that myeloma cells, unlike normal plasma cells, exhibit high levels of endogenous DNA damage, evidenced by the presence of γH2AX foci [36].

Increased endogenous formation of DNA damage and/or decreased DNA repair effectiveness are two possible, though not exclusive, processes that can contribute to the accumulation of DNA damage. In this study, we assessed redox dysregulation and apurinic/apyrimidinic sites at baseline, two important endogenous factors that result in the intracellular generation of SSBs and DSBs. Our results demonstrated that MM1S and OPM2 cell lines showed the lowest GSH/GSSG ratio and the highest AP-sites, while on the contrary AMO1 and XG-6 the highest GSH/GSSG ratio and the lowest AP-sites. Accumulating evidence indicates that, because of oncogene activation and/or increased metabolic activity, cancer cells generate more ROS than healthy cells [37,38]. It has been demonstrated that the increased ROS levels in these cells augment invasion, motility, and proliferation [37]. MM cells generate ROS from several sources, such as the mitochondria, the NADPH oxidases, and the endoplasmic reticulum [18,20]. Interestingly, since in these cells elevated immunoglobulin synthesis is an additional process that exacerbates ROS overproduction [39], further enhancement of oxidative stress may serve as an effective approach to address this disease. Moreover, in MM progression there is a notable imbalance in the patient’s serum, characterized by a depletion of antioxidants and an increase in pro-oxidant molecules. This shift towards a more pro-oxidative environment is linked to the disease’s advancement and severity [19,22]. Indeed, in MM patients, serum levels of antioxidants, like vitamins C and E, glutathione peroxidase, catalase, and superoxide dismutase, were reduced, while oxidative stress markers were elevated compared to healthy individuals [22].

Another key source of endogenous/baseline DNA damage is the presence of apurinic/apyrimidinic sites. These are common forms of DNA lesions that occur at rates of 10,000 to 30,000 per human cell per day. Previous studies have shown that oxidative stress can result in the generation of apurinic/apyrimidinic sites [40]. In fact, AP-sites are formed when the N-glycosylic bond linking a damaged or modified base (such as an oxidized, alkylated, or deaminated purine) to the DNA sugar–phosphate backbone is broken, either spontaneously or by the action of DNA glycosylases. Cleavage of apurinic/apyrimidinic lesions by AP endonucleases or DNA N-glycosylases/AP lyases results in the induction of DNA SSBs and DSBs [41]. Notably, the correlation between basal DNA damage, redox dysregulation, and AP-sites has also been reported in several other pathological conditions like lung cancer [42] and head and neck carcinoma [43], as well as in autoimmune diseases such as rheumatoid arthritis [44] and systemic sclerosis [45].

Next, the efficiencies of critical DNA repair mechanisms, namely NER, ICL repair, and DSB repair, were also analyzed. To trigger NER, cells were irradiated with UVC, which directly induces DNA lesions, forming CPDs and (6-4)PPs [46]. To measure these lesions, we used the alkaline comet assay that determines SSBs, DSBs, and alkali-labile sites (ALs), which include apurinic/apyrimidinic sites, as these ALs are converted to SSBs under the high-pH denaturing conditions of the assay [47]. Previous studies have shown that by irradiating cells with UVC and then monitoring the DNA damage repair kinetics over a 6 h period, comet assay quantifies the cells’ ability to remove these lesions, thus providing an indirect determination of their NER capacity [48,49,50,51]. Moreover, to trigger ICL repair and DNA DSB repair mechanisms, cells were treated with 100 μg/mL melphalan for 5 min, and melphalan-induced adducts were measured. That is, using Southern blot, ICLs were measured at the active *N-ras* gene, the repair rate of which correlates with melphalan-induced apoptosis [52,53]. Southern blotting is an indirect method for determining ICL repair, since it does not directly measure ICL lesions, but rather the effects of their removal on DNA fragments over time. Also, measurement of γH2AX foci was used as a sensitive, indirect marker for DNA DSBs because they are formed rapidly and specifically at the sites of DSBs in response to DNA damage [54]. In addition, we measured RAD51 foci, a functional marker for HR repair, allowing for the identification of tumors with intact HR pathways. Tumors with functional HR use RAD51 to repair DNA damage, making them resistant to treatments that damage DNA [55].

In line with the results presented above, MM1S and OPM2 exhibited the lowest NER and ICL repair capacities, while AMO1 and XG-6 exhibited the highest ones. NER is an important mechanism that removes a wide range of bulky DNA lesions, such as those produced by UV light, ROS, endogenous lipid peroxidation products, environmental mutagens, smoking-associated carcinogens, and chemotherapeutic agents, such as melphalan [56]. The genes XPC (xeroderma pigmentosum, complementation group C) and ERCC3 (XPB) are particularly relevant to NER in MM. In fact, studies have shown that knocking down these genes increases the sensitivity of myeloma cells to melphalan [56]. Notably, it is demonstrated that the inhibition of NER pathways can overcome resistance to alkylating agents in myeloma cells. Specifically, inhibiting the XPB protein can simultaneously inhibit both NER and transcription, making it a potentially effective therapeutic strategy. The efficiency of ICL repair was also evaluated. ICLs are covalent bonds that are formed between two complementary strands of DNA, effectively preventing DNA replication and transcription [57]. These toxic DNA lesions can be caused by certain chemotherapeutic drugs like melphalan and by endogenous sources like lipid peroxidation and are involved in the treatment of MM [58]. The repair of these crosslinks is crucial for cell survival, and in cancer cells it can contribute to drug resistance. DSB repair dynamics were assessed via quantification of γH2AX and RAD51 foci formation following melphalan treatment. No significant differences in the accumulation of γH2AX between the HMCLs were observed, with most cell lines showing a DSB repair deficient phenotype and impaired or delayed RAD51 foci induction, suggesting a deregulated HR pathway. In line with these data, previous studies have shown that in MM, DSB repair pathways are frequently deregulated, thus contributing to the disease’s genomic instability [59,60]. Notably, the two main pathways for repairing DSBs, namely NHEJ and HR, often exhibit altered activities in MM [59,61].

Chromatin condensation was also analyzed. In line with the NER capacity, MM1S and OPM2 showed the lowest chromatin looseness, while AMO1 and XG-6 showed the highest ones. Chromatin structure plays a significant role in MM by influencing gene expression, drug sensitivity, and disease progression [30,62]. Alterations in histone modifications and DNA methylation can lead to aberrant gene expression patterns that promote myeloma cell growth and survival. Furthermore, chromatin structure can affect the efficiency of DNA repair mechanisms, which in turn impacts how myeloma cells respond to chemotherapy. Chromatin remodeling complexes, like the SWI/SNF complex, are frequently mutated in MM, indicating their importance in disease progression. These mutations can disrupt normal gene expression patterns and contribute to uncontrolled cell growth [62]. Also, aberrant histone modifications in myeloma cells can lead to the upregulation of oncogenes or the downregulation of tumor suppressor genes, contributing to disease development and progression [63,64]. Moreover, changes in chromatin structure can affect the accessibility of DNA to antimyeloma drugs, influencing their effectiveness [30]. Notably, changes in chromatin structure have been observed in both PBMCs and BMPCs during myelomagenesis.

We acknowledge that our study has limitations. First, the analysis is limited only to cell lines. Therefore, a key challenge for our future research is to examine redox status/DDR signals in primary samples (PBMCs and BMPCs) from MM patients, an approach that would strengthen translational relevance. Second, no mutational or transcriptomic profiling accompanies the DDR phenotypes. In fact, it would provide mechanistic depth to correlate DDR defects with known MM driver mutations (such as those in the TP53, ATM, and BRCA pathways). Third, we used classical methodologies, such as comet assay and γH2AX fluorescence detection. Complementary methods (e.g., reporter assays for HR/NHEJ) could validate the findings.

The differences in DNA repair capacity among MM cell lines highlight the significance of DDR inhibitors as a therapeutic strategy. By inhibiting DDR components, these drugs can disrupt the ability of cancer cells to repair DNA damage, leading to increased genomic instability, accumulation of mutations, and ultimately cell death, particularly when combined with DNA-damaging agents or radiotherapy. Inhibiting DDR components, like PARP, ATR, WEE1, CHK1/2, ATM, and DNA-PKs, is under evaluation in early-phase studies for multiple myeloma and other hematological malignancies showing promising preliminary signals of activity [65,66,67]. In addition, recent data reveal that inhibiting other DDR-associated targets, such as histone lysine demethylases (LSD1, KDM4, KDM6) might be a promising therapeutic strategy for cancer, offering improved patient outcomes [68,69,70].

Together, the results presented herein showed that DDR parameters and redox status correlate with the sensitivity of MM cells to genotoxic treatment, specifically melphalan. Interestingly, instead of using single-line models, the work adds value by conducting a comparative panel study of eleven MM cell lines plus a healthy control and showing that apoptosis susceptibility is correlated with baseline DNA damage, GSH/GSSG ratio, AP-sites, and impaired repair capacity. The topic is extremely topical and important. Indeed, one of the biggest clinical challenges in MM is chemotherapy resistance. Although oxidative stress and DDR have been thoroughly investigated separately, little is known about how they interact to influence treatment response in MM cell lines. By systematically combining DDR profiling, chromatin structure, and redox status, this study addresses a clear gap.

## 4. Materials and Methods

### 4.1. Cell Lines

Human MM cell lines U266 and NCI-H929 were purchased from the American Type Culture Collection (ATCC, Manassas, VA, USA); RPMI-8226, OPM2, LP1, MM1S, AMO1, SKMM2, XG-1, XG-6, and XG-7 were kindly provided by Prof. J. Moreaux (University of Montpellier, Montpellier, France). All HMCLs were cultured in RPMI1640 medium, supplemented with 20% Fetal bovine serum (FBS) and 1% penicillin/streptomycin. For the IL-6 dependent cell lines (XG-1, XG-6 and XG-7) 2 ng/mL of IL-6 was added. The immortalized healthy B lymphoblastoid cell line RPMI-1788 was purchased by Cytion (CLS Cell Lines Service GmbH, Eppelheim, Germany, #300318) and cultured in RPMI1640 medium, supplemented with 20% FBS and 1% penicillin/streptomycin.

### 4.2. MTT Viability Assay

Cell viability was examined 6 h and 24 h following UVC irradiation. A total of 1 × 10^6^ cells/mL in PBS (Phosphate-Buffered Saline) suspension was irradiated (0–100 J/m^2^) in 6-well plates, harvested, and viability was examined using MTT assay [71]. Briefly, 1 × 10^4^ treated cells were seeded on 96-well plates and incubated with 0.5 mg/mL MTT (Thermo Fisher Scientific, Waltham, MA, USA, #M6494) for 3.5 h. Solubilization step then followed with Dimethyl Sulfoxide (DMSO) as solvent (AppliChem, Darmstadt, Germany, #A3672) for 15 min and dye’s absorbance was measured at 570 nm with a reference wavelength of 690 nm on a Tecan Safire2 microplate reader (Tecan, Männedorf, Switzerland).

### 4.3. Measurement of NER

To assess the efficiency of NER, suspension of 1 × 10^6^ cells/mL in PBS was seeded in 6-well plates and irradiated with UVC (50 J/m^2^). Then, the medium changed to the appropriate one and the samples were incubated for 0–6 h at 37 °C, harvested, and analyzed using alkaline comet assay [43]. Briefly, 1 × 10^4^ cells in 1% low-melting-point agarose were spread onto microscope slides and lysed for 2 h at 4 °C in alkaline solution (2.5 M NaCl, 0.1 M EDTA, 0.01 M Tris; pH 10, 1% Triton X-100). Electrophoresis was performed for 30 min (25 V, 225 mA). Samples were stained with SYBR^TM^ Gold Nucleic Acid Gel Stain (Thermo Fischer Scientific, Waltham, MA, USA, #S11494) and imaged with a fluorescence microscope using 10× lens Zeiss Axiophot (Zeiss, Oberkochen, Germany). Comet parameters were analyzed by the ImageJ Analysis module (Version 1.54p), Open Comet (Version 1.3.1., https://cometbio.org/, accessed on 18 October 2025). Olive Tail Moment (OTM) for each sample was calculated by a total average of >200 cells.

### 4.4. Measurement of ICL Repair

Suspension of 1 × 10^6^ cells/mL was treated with melphalan (100 μg/mL for 5 min) in their appropriate medium at 37 °C, changed to drug-free medium for 0–48 h, harvested, and ICLs were measured in the *N-ras* gene using Southern blot [42]. Briefly, genomic DNA was isolated, digested with the restriction enzyme EcoRI, and denatured in 50 mM NaOH for 15 min at 37 °C. Denaturation step was then stopped on ice and samples were mixed with loading buffer (0.2% Ficoll, 0.1 mM EDTA, 0.01% bromocresol green). Electrophoresis and hybridization were performed for 16 h at 30 V in 0.6% agarose gel with 40 mM Tris-acetate and 2 mM EDTA. The number of ICLs per restriction fragment was calculated using the Poisson distribution formula: [Crosslinks per fragment = −log_e_(fraction of fragments free of crosslinks)].

### 4.5. Measurement of DSB Repair

Cell lines (1 × 10^6^ cells/mL) were treated with 100 μg/mL melphalan for 5 min in the appropriate medium, changed to drug-free medium for 0–24 h, harvested, and aliquots containing 5 × 10^5^ cells were adhered to coverslips and fixed with ice cold 4% paraformaldehyde for 15 min. Permeabilization step followed for 10 min [0.25% Triton-X in Phosphate-buffered saline (PBS)] and then nonspecific binding was eliminated with a 30 min incubation in blocking buffer [1% Bovine serum albumin (BSA), 0.25% Triton in PBS]. Cells were incubated with primary antibody against γH2AX (Cell signaling, Danvers, MA, USA, #80312; 1:400 for 1 h) and RAD51 (Cell signaling, #65653; 1:400 for 1 h) followed by fluorescent secondary antibody incubation with Alexa Fluor 488 (Invitrogen, Waltham, MA, USA, #481679) and Alexa Fluor 568 (Invitrogen, #453569) at 1:1000 for 1 h. Imaging was performed on a confocal scanning microscope (Leica TCS SP-1, Leica Microsystems CMS GmbH, Mannheim, Germany). The γH2AX and RAD51 foci were analyzed using open access software (FoCo, MATLAB, https://sourceforge.net/projects/focicount/, accessed on 18 October 2025) [72]. At least 100 cells per treatment were analyzed and each experiment was performed in triplicate.

### 4.6. Oxidative Stress and Apurinic/Apyrimidinic Sites Assessment

To measure oxidative stress, the use of GSH/GSSG ratio was assessed via a luminescence-based assay following the manufacturer’s protocol (GSH/GSSG-Glo Assay, Promega, Madison, WI, USA, #V6612). A total of 1 × 10^4^ cells were used per condition. For the quantification of apurinic/apyrimidinic (AP, abasic) sites, samples were analyzed with the OxiSelect Oxidative DNA Damage Quantification Kit (Cell Biolabs, San Diego, CA, USA; #STA-324) according to the manufacturer’s protocol.

### 4.7. Apoptosis Rates

A total of 1 × 10^6^ cells/mL were treated with increasing doses of melphalan (0–100 μg/mL, 5 min, 37 °C) in the appropriate medium. Then, 1 × 10^4^ cells per well were seeded in 96-well plates and incubated for 24 h in melphalan-free medium. Apoptosis was assessed using the Cell Death Detection ELISA PLUS kit (Roche Diagnostics Corp., Basel, Switzerland, #11774425001) following the manufacturer’s protocol.

### 4.8. Measurement of Chromatin Condensation

Nuclear isolation and micrococcal nuclease digestion were carried out using standard procedures [73]. In brief, hypotonic buffer (10 mM Tris–HCl, pH 8.0, 10 mM NaCl, 5 mM MgCl_2_) was used to swell 5 × 10^6^ cells for 30 min at 4 °C. The cells were homogenized in hypotonic buffer containing 0.3% Nonidet P-40, the nuclei were purified by centrifugation (1500× *g* for 10 min) through hypotonic buffer containing 8.5% sucrose (*w*/*v*), and then resuspended in digestion buffer (100 mM Tris–HCl, pH 8.0, 50 mM NaCl, 3 mM MgCl_2_, 1 mM CaCl_2_). Micrococcal nuclease (1U) was used to immediately digest the isolated nuclei at 37 °C for 5 min. An equal volume of stop solution (200 mM Tris–HCl, pH 8.0, 200 mM NaCl, 20 mM EDTA, 2% SDS, 200 μg/mL proteinase K) was added to terminate the digestion. Genomic DNA was purified and then electrophoretically separated in 1.5% agarose gels. DNA was transferred to nitrocellulose (Amersham Hybond-N+, Cytiva, Marlborough, MA, USA; RPN303B) and was later hybridized to probes specific for the *N-ras* gene [73].

### 4.9. Statistical Analysis

Analyses were performed using SPSS software (SPSS for Windows, version 30.0, SPSS Inc., Chicago, IL, USA). The relationship between markers of the cell lines was explored by using Pearson correlation matrix. Clustering of the markers was assigned by two step analysis. Principal component analysis (PCA) was conducted to generate a pattern of measured markers for the cell lines. For further investigation, hierarchical cluster analysis was carried out by using Z scores of the markers and Ward method to equalize the size of the clusters between the cell lines. As the hierarchical cluster analysis indicated, we categorized the cell lines by creating a three-level categorical variable. The three groups were: Group A (MM1S and OPM2), Group B (LP1, RPMI-8226, and SKMM2) and Group C (RPMI-1788, U266, AMO1, NCI-H929, XG-1, XG-6, and XG-7). Finally, we operated one-way ANOVA statistical test to identify the differences among the markers in terms of grouping functionality of the cell lines. Post hoc analysis was performed with Bonferroni correction (to reduce the risk of making a Type I error) and *p*-values < 0.05 were considered as significant. Graph design was carried out with GraphPad Prism 8.0.1. The mean ± SD was used to present the data.

## 5. Conclusions

In this study, we performed a comprehensive analysis of functional DDR-associated aberrations across a panel of human MM cell lines with different sensitivities to genotoxic insults. By integrating assays for basal DNA damage, redox status, accumulation of apurinic/apyrimidinic lesions, DNA repair efficiencies, chromatin structure, and apoptosis rates, we uncovered distinct DDR competency amongst the cell lines under study. We found that HMCLs with increased susceptibility to genotoxic agents exhibited elevated basal DNA damage, reduced GSH/GSSG ratio, deficient DNA repair capacity, and more condensed chromatin structure. These results highlight the interplay between redox status and DNA repair efficiency, underscoring the DDR dysfunction as both an important factor of MM pathophysiology and a determinant of therapeutic vulnerability. Incorporating functional redox status/DDR profiling in MM research may contribute to the development of new effective biomarkers.

## Figures and Tables

**Table 1:** Pearson correlation analysis among DDR-related parameters in MM cell lines.

	Apoptosis Rates	DNA Damage	GSH/GSSG Ratio	AP-Sites	NER Capacity (AUC)	ICL/R Capacity (AUC)	γH2AX Foci (AUC)
**DNA damage**	−0.918 **						
**GSH/GSSG ratio**	0.792 **	−0.804 **					
**AP-sites**	−0.864 **	0.864 **	−0.814 **				
**NER Capacity (AUC) ^#^**	−0.792 **	0.762 **	−0.648 *	0.892 **			
**ICL/R Capacity (AUC)**	−0.617 *	0.456	−0.485	0.592 *	0.707 *		
**γH2AX foci (AUC)**	0.135	−0.145	−0.053	0.021	−0.003	0.483	
**RAD51 foci (AUC)**	−0.032	−0.033	0.286	−0.215	−0.244	0.073	−0.03

** Correlation is significant at the 0.01 level (2-tailed); * Correlation is significant at the 0.05 level (2-tailed); ^#^ AUC: Area Under the Curve.

## Data Availability

The original contributions presented in this study are included in the article/Supplementary Material. Further inquiries can be directed to the corresponding author.

## Supplementary Materials

The following supporting information can be downloaded at https://www.mdpi.com/article/10.3390/ijms262010171/s1.

## References

[1] Saade C., Ghobrial I.M. (2025). Updates on Mechanisms of Disease Progression in Precursor Myeloma: Monoclonal Gammopathy of Undermined Significance and Smoldering Myeloma. Presse Méd..

[2] Kyle R.A., Larson D.R., Therneau T.M., Dispenzieri A., Kumar S., Cerhan J.R., Rajkumar S.V. (2018). Long-Term Follow-up of Monoclonal Gammopathy of Undetermined Significance. N. Engl. J. Med..

[3] Rajkumar S.V. (2022). Multiple Myeloma: 2022 Update on Diagnosis, Risk Stratification, and Management. Am. J. Hematol..

[4] Kazandjian D. (2016). Multiple Myeloma Epidemiology and Survival: A Unique Malignancy. Semin. Oncol..

[5] Ludwig H., Durie S.N., Meckl A., Hinke A., Durie B. (2020). Multiple Myeloma Incidence and Mortality Around the Globe; Interrelations Between Health Access and Quality, Economic Resources, and Patient Empowerment. Oncologist.

[6] Fonseca R., Abouzaid S., Bonafede M., Cai Q., Parikh K., Cosler L., Richardson P. (2017). Trends in Overall Survival and Costs of Multiple Myeloma, 2000–2014. Leukemia.

[7] Morgan G.J., Walker B.A., Davies F.E. (2012). The Genetic Architecture of Multiple Myeloma. Nat. Rev. Cancer.

[8] Malamos P., Papanikolaou C., Gavriatopoulou M., Dimopoulos M.A., Terpos E., Souliotis V.L. (2024). The Interplay between the DNA Damage Response (DDR) Network and the Mitogen-Activated Protein Kinase (MAPK) Signaling Pathway in Multiple Myeloma. Int. J. Mol. Sci..

[9] Elbezanti W.O., Challagundla K.B., Jonnalagadda S.C., Budak-Alpdogan T., Pandey M.K. (2023). Past, Present, and a Glance into the Future of Multiple Myeloma Treatment. Pharmaceuticals.

[10] Sousa M.M.L., Zub K.A., Aas P.A., Hanssen-Bauer A., Demirovic A., Sarno A., Tian E., Liabakk N.B., Slupphaug G. (2013). An Inverse Switch in DNA Base Excision and Strand Break Repair Contributes to Melphalan Resistance in Multiple Myeloma Cells. PLoS ONE.

[11] Zub K.A., Sousa M.M.L.d., Sarno A., Sharma A., Demirovic A., Rao S., Young C., Aas P.A., Ericsson I., Sundan A. (2015). Modulation of Cell Metabolic Pathways and Oxidative Stress Signaling Contribute to Acquired Melphalan Resistance in Multiple Myeloma Cells. PLoS ONE.

[12] Petrilla C., Galloway J., Kudalkar R., Ismael A., Cottini F. (2023). Understanding DNA Damage Response and DNA Repair in Multiple Myeloma. Cancers.

[13] Besse A., Besse L., Kraus M., Mendez-Lopez M., Bader J., Xin B.-T., Bruin G.d., Maurits E., Overkleeft H.S., Driessen C. (2019). Proteasome Inhibition in Multiple Myeloma: Head-to-Head Comparison of Currently Available Proteasome Inhibitors. Cell Chem. Biol..

[14] Holstein S.A., McCarthy P.L. (2017). Immunomodulatory Drugs in Multiple Myeloma: Mechanisms of Action and Clinical Experience. Drugs.

[15] Sammartano V., Franceschini M., Fredducci S., Caroni F., Ciofini S., Pacelli P., Bocchia M., Gozzetti A. (2023). Anti-BCMA Novel Therapies for Multiple Myeloma. Cancer Drug Resist..

[16] Parikh R.H., Lonial S. (2023). Chimeric Antigen Receptor T-Cell Therapy in Multiple Myeloma: A Comprehensive Review of Current Data and Implications for Clinical Practice. CA Cancer J. Clin..

[17] Devarakonda S., Efebera Y., Sharma N. (2021). Role of Stem Cell Transplantation in Multiple Myeloma. Cancers.

[18] Lipchick B.C., Fink E.E., Nikiforov M.A. (2016). Oxidative Stress and Proteasome Inhibitors in Multiple Myeloma. Pharmacol. Res..

[19] Athanasopoulou S., Kapetanou M., Magouritsas M.G., Mougkolia N., Taouxidou P., Papacharalambous M., Sakellaridis F., Gonos E. (2022). Antioxidant and Antiaging Properties of a Novel Synergistic Nutraceutical Complex: Readouts from an In Cellulo Study and an In Vivo Prospective, Randomized Trial. Antioxidants.

[20] Caillot M., Dakik H., Mazurier F., Sola B. (2021). Targeting Reactive Oxygen Species Metabolism to Induce Myeloma Cell Death. Cancers.

[21] Xiong S., Chng W.-J., Zhou J. (2021). Crosstalk between Endoplasmic Reticulum Stress and Oxidative Stress: A Dynamic Duo in Multiple Myeloma. Cell. Mol. Life Sci..

[22] Kul A.N., Kurt B.O. (2024). Multiple Myeloma from the Perspective of Pro- and Anti-Oxidative Parameters: Potential for Diagnostic and/or Follow-Up Purposes?. J. Pers. Med..

[23] Chatterjee N., Walker G.C. (2017). Mechanisms of DNA Damage, Repair, and Mutagenesis. Environ. Mol. Mutagen..

[24] O’Connor M.J. (2015). Targeting the DNA Damage Response in Cancer. Mol. Cell.

[25] Saitoh T., Oda T. (2021). DNA Damage Response in Multiple Myeloma: The Role of the Tumor Microenvironment. Cancers.

[26] Alagpulinsa D.A., Szalat R.E., Poznansky M.C., Reis R.J.S. (2020). Genomic Instability in Multiple Myeloma. Trends Cancer.

[27] Sharma A., Heuck C.J., Fazzari M.J., Mehta J., Singhal S., Greally J.M., Verma A. (2010). DNA Methylation Alterations in Multiple Myeloma as a Model for Epigenetic Changes in Cancer. Wiley Interdiscip. Rev. Syst. Biol. Med..

[28] Feng Z., Hu W., Chasin L.A., Tang M. (2003). Effects of Genomic Context and Chromatin Structure on Transcription-coupled and Global Genomic Repair in Mammalian Cells. Nucleic Acids Res..

[29] Papanikolaou C., Economopoulou P., Spathis A., Kotsantis I., Gavrielatou N., Anastasiou M., Moutafi M., Kyriazoglou A., Foukas G.-R.P., Lelegiannis I.M. (2025). Association of DNA Damage Response Signals and Oxidative Stress Status with Nivolumab Efficacy in Patients with Head and Neck Squamous Cell Carcinoma. Br. J. Cancer.

[30] Gkotzamanidou M., Terpos E., Bamia C., Kyrtopoulos S.A., Sfikakis P.P., Dimopoulos M.A., Souliotis V.L. (2014). Progressive Changes in Chromatin Structure and DNA Damage Response Signals in Bone Marrow and Peripheral Blood during Myelomagenesis. Leukemia.

[31] Xu X., Lai Y., Hua Z.-C. (2019). Apoptosis and Apoptotic Body: Disease Message and Therapeutic Target Potentials. Biosci. Rep..

[32] Enoiu M., Jiricny J., Schärer O.D. (2012). Repair of Cisplatin-Induced DNA Interstrand Crosslinks by a Replication-Independent Pathway Involving Transcription-Coupled Repair and Translesion Synthesis. Nucleic Acids Res..

[33] Souliotis V.L., Dimopoulos M.A., Sfikakis P.P. (2003). Gene-Specific Formation and Repair of DNA Monoadducts and Interstrand Cross-Links after Therapeutic Exposure to Nitrogen Mustards. Clin. Cancer Res..

[34] Herrero A.B., Gutiérrez N.C. (2017). Targeting Ongoing DNA Damage in Multiple Myeloma: Effects of DNA Damage Response Inhibitors on Plasma Cell Survival. Front. Oncol..

[35] Kumar S., Gkotzamanidou M., Talluri S., Day M., Neptune M., Potluri L.B., Chakraborty C., Mills K., Shammas M.A., Munshi N.C. (2019). Ongoing Spontaneous DNA Damage Creates Synthetic Lethality Targeted By Novel RAD51 Inhibitors in Multiple Myeloma. Blood.

[36] Walters D.K., Wu X., Tschumper R.C., Arendt B.K., Huddleston P.M., Henderson K.J., Dispenzieri A., Jelinek D.F. (2011). Evidence for Ongoing DNA Damage in Multiple Myeloma Cells as Revealed by Constitutive Phosphorylation of H2AX. Leukemia.

[37] Nakamura H., Takada K. (2021). Reactive Oxygen Species in Cancer: Current Findings and Future Directions. Cancer Sci..

[38] Attique I., Haider Z., Khan M., Hassan S., Soliman M.M., Ibrahim W.N., Anjum S. (2025). Reactive Oxygen Species: From Tumorigenesis to Therapeutic Strategies in Cancer. Cancer Med..

[39] Wang J., Lin D., Peng H., Huang Y., Huang J., Gu J. (2013). Cancer-Derived Immunoglobulin G Promotes Tumor Cell Growth and Proliferation through Inducing Production of Reactive Oxygen Species. Cell Death Dis..

[40] Yuan H.-H., Yin H., Marincas M., Xie L.-L., Bu L.-L., Guo M.-H., Zheng X.-L. (2025). From DNA Repair to Redox Signaling: The Multifaceted Role of APEX1 (Apurinic/Apyrimidinic Endonuclease 1) in Cardiovascular Health and Disease. Int. J. Mol. Sci..

[41] Thompson P.S., Cortez D. (2020). New Insights into Abasic Site Repair and Tolerance. DNA Repair.

[42] Mavroeidi D., Georganta A., Stefanou D.T., Papanikolaou C., Syrigos K.N., Souliotis V.L. (2024). DNA Damage Response Network and Intracellular Redox Status in the Clinical Outcome of Patients with Lung Cancer. Cancers.

[43] Papanikolaou C., Economopoulou P., Gavrielatou N., Mavroeidi D., Psyrri A., Souliotis V.L. (2025). UVC-Induced Oxidative Stress and DNA Damage Repair Status in Head and Neck Squamous Cell Carcinoma Patients with Different Responses to Nivolumab Therapy. Biology.

[44] Souliotis V.L., Vlachogiannis N.I., Pappa M., Argyriou A., Ntouros P.A., Sfikakis P.P. (2020). DNA Damage Response and Oxidative Stress in Systemic Autoimmunity. Int. J. Mol. Sci..

[45] Vlachogiannis N.I., Pappa M., Ntouros P.A., Nezos A., Mavragani C.P., Souliotis V.L., Sfikakis P.P. (2020). Association Between DNA Damage Response, Fibrosis and Type I Interferon Signature in Systemic Sclerosis. Front. Immunol..

[46] Rastogi R.P., Richa, Kumar A., Tyagi M.B., Sinha R.P. (2010). Molecular Mechanisms of Ultraviolet Radiation-Induced DNA Damage and Repair. J. Nucleic Acids.

[47] Collins A., Møller P., Gajski G., Vodenková S., Abdulwahed A., Anderson D., Bankoglu E.E., Bonassi S., Boutet-Robinet E., Brunborg G. (2023). Measuring DNA Modifications with the Comet Assay: A Compendium of Protocols. Nat. Protoc..

[48] Zheng W., He J.-L., Jin L.-F., Lou J.-L., Wang B.-H. (2005). Assessment of Human DNA Repair (NER) Capacity with DNA Repair Rate (DRR) by Comet Assay. Biomed. Environ. Sci..

[49] Souliotis V.L., Vlachogiannis N.I., Pappa M., Argyriou A., Sfikakis P.P. (2019). DNA Damage Accumulation, Defective Chromatin Organization and Deficient DNA Repair Capacity in Patients with Rheumatoid Arthritis. Clin. Immunol..

[50] Stefanou D.T., Kouvela M., Stellas D., Voutetakis K., Papadodima O., Syrigos K., Souliotis V.L. (2022). Oxidative Stress and Deregulated DNA Damage Response Network in Lung Cancer Patients. Biomedicines.

[51] Pappa M., Ntouros P.A., Papanikolaou C., Sfikakis P.P., Souliotis V.L., Tektonidou M.G. (2023). Augmented Oxidative Stress, Accumulation of DNA Damage and Impaired DNA Repair Mechanisms in Thrombotic Primary Antiphospholipid Syndrome. Clin. Immunol..

[52] Dimopoulos M.A., Souliotis V.L., Anagnostopoulos A., Papadimitriou C., Sfikakis P.P. (2005). Extent of Damage and Repair in the P53 Tumor-Suppressor Gene After Treatment of Myeloma Patients with High-Dose Melphalan and Autologous Blood Stem-Cell Transplantation Is Individualized and May Predict Clinical Outcome. J. Clin. Oncol..

[53] Gkotzamanidou M., Terpos E., Bamia C., Munshi N.C., Dimopoulos M.A., Souliotis V.L. (2016). DNA Repair of Myeloma Plasma Cells Correlates with Clinical Outcome: The Effect of the Nonhomologous End-Joining Inhibitor SCR7. Blood.

[54] Prabhu K.S., Kuttikrishnan S., Ahmad N., Habeeba U., Mariyam Z., Suleman M., Bhat A.A., Uddin S. (2024). H2AX: A Key Player in DNA Damage Response and a Promising Target for Cancer Therapy. Biomed. Pharmacother..

[55] Cruz C., Castroviejo-Bermejo M., Gutiérrez-Enríquez S., Llop-Guevara A., Ibrahim Y.H., Gris-Oliver A., Bonache S., Morancho B., Bruna A., Rueda O.M. (2018). RAD51 Foci as a Functional Biomarker of Homologous Recombination Repair and PARP Inhibitor Resistance in Germline BRCA-Mutated Breast Cancer. Ann. Oncol..

[56] Szalat R., Samur M.K., Fulciniti M., Lopez M., Nanjappa P., Cleynen A., Wen K., Kumar S., Perini T., Calkins A.S. (2018). Nucleotide Excision Repair Is a Potential Therapeutic Target in Multiple Myeloma. Leukemia.

[57] Deans A.J., West S.C. (2011). DNA Interstrand Crosslink Repair and Cancer. Nat. Rev. Cancer.

[58] Shukla P., Solanki A., Ghosh K., Vundinti B.R. (2013). DNA Interstrand Cross-Link Repair: Understanding Role of Fanconi Anemia Pathway and Therapeutic Implications. Eur. J. Haematol..

[59] Herrero A.B., Miguel J.S., Gutierrez N.C. (2015). Deregulation of DNA Double-Strand Break Repair in Multiple Myeloma: Implications for Genome Stability. PLoS ONE.

[60] Gourzones-Dmitriev C., Kassambara A., Sahota S., Rème T., Moreaux J., Bourquard P., Hose D., Pasero P., Constantinou A., Klein B. (2013). DNA Repair Pathways in Human Multiple Myeloma. Cell Cycle.

[61] Gillyard T., Davis J., Weyemi U., Galluzzi L. (2021). Chapter Two—DNA Double-Strand Break Repair in Cancer: A Path to Achieving Precision Medicine. International Review of Cell and Molecular Biology.

[62] Chakraborty C., Mukherjee S. (2022). Molecular Crosstalk between Chromatin Remodeling and Tumor Microenvironment in Multiple Myeloma. Curr. Oncol..

[63] Ohguchi H., Hideshima T., Anderson K.C. (2018). The Biological Significance of Histone Modifiers in Multiple Myeloma: Clinical Applications. Blood Cancer J..

[64] Tigu A.B., Ivancuta A., Uhl A., Sabo A.C., Nistor M., Mureșan X.-M., Cenariu D., Timis T., Diculescu D., Gulei D. (2025). Epigenetic Therapies in Melanoma—Targeting DNA Methylation and Histone Modification. Biomedicines.

[65] De Mel S., Lee A.R., Tan J.H.I., Tan R.Z.Y., Poon L.M., Chan E., Lee J., Chee Y.L., Lakshminarasappa S.R., Jaynes P.W. (2024). Targeting the DNA Damage Response in Hematological Malignancies. Front. Oncol..

[66] Kwok M., Agathanggelou A., Stankovic T. (2024). DNA Damage Response Defects in Hematologic Malignancies: Mechanistic Insights and Therapeutic Strategies. Blood.

[67] Shim Y.J. (2025). Therapeutic Targeting of DNA Damage Response Pathways in TP53- and ATM-Mutated Tumors. Brain Tumor Res. Treat..

[68] Bandini C., Mereu E., Paradzik T., Labrador M., Maccagno M., Cumerlato M., Oreglia F., Prever L., Manicardi V., Taiana E. (2023). Lysin (K)-Specific Demethylase 1 Inhibition Enhances Proteasome Inhibitor Response and Overcomes Drug Resistance in Multiple Myeloma. Exp. Hematol. Oncol..

[69] Rao D., Wang Y., Yang X., Chen Z., Wu F., Ren R., Sun Y., Lai Y., Peng L., Yu L. (2025). Discovery of a First-in-Class PROTAC Degrader of Histone Lysine Demethylase KDM4. Eur. J. Med. Chem..

[70] Nastaranpour M., Damara A., Grabbe S., Shahneh F. (2025). Lysine Demethylase 6 (KDM6): A Promising Therapeutic Target in Autoimmune Disorders and Cancer. Biomed. Pharmacother..

[71] Avramis I.A., Christodoulopoulos G., Suzuki A., Laug W.E., Gonzalez-Gomez I., McNamara G., Sausville E.A., Avramis V.I. (2002). In Vitro and in Vivo Evaluations of the Tyrosine Kinase Inhibitor NSC 680410 against Human Leukemia and Glioblastoma Cell Lines. Cancer Chemother. Pharmacol..

[72] Lapytsko A., Kollarovic G., Ivanova L., Studencka M., Schaber J. (2015). FoCo: A Simple and Robust Quantification Algorithm of Nuclear Foci. BMC Bioinform..

[73] Souliotis V.L., Dimopoulos M.A., Episkopou H.G., Kyrtopoulos S.A., Sfikakis P.P. (2006). Preferential In Vivo DNA Repair of Melphalan-Induced Damage in Human Genes Is Greatly Affected by the Local Chromatin Structure. DNA Repair.

