# Supplementary material for: DNA Damage Response and Redox Status in the Resistance of Multiple Myeloma Cells to Genotoxic Treatment
Source: Int J Mol Sci. 2025 Oct 19;26(20):10171. doi: 10.3390/ijms262010171 (PMC12563694; doi:10.3390/ijms262010171)
Supplement: Supplementary file 1 [file ijms-26-10171-s001.zip › ijms-3887103-supplementary.pdf]

Supplementary Materials

DNA damage response and oxidative stress in the resistance of multiple myeloma cells to genotoxic treatment

Malamos P. et al.

Table of Contents

Supplementary Table S1. ANOVA results on DDR related parameters among the three groups post hierarchical clustering analysis

Supplementary Figure S1. UVC-induced sensitivity

Supplementary Figure S2. UVC-induced DDR signals in MM cell lines

Supplementary Figure S3. DDR-related signals in MM cells following melphalan treatment

Supplementary Figure S4. One-way ANOVA results on the DDR and redox markers among the three groups generated from clustering analysis

Supplementary Table S1. ANOVA results on DDR related parameters among the three groups post hierarchical clustering analysis

Markers	Mean (± SD)			p values		
	Group A	Group B	Group C	Group A vs Group B	Group A vs Group C	Group B vs Group C
**Apoptosis rates [Dose inducing apoptosis (μg/ml)]**	15 (± 1.761167)	40.41667 (± 11.814539)	61.82143 (± 7.532865)	0.006	< 0.001	0.003
**Endogenous/baseline DNA Damage (OTM; Arbitrary units)**	12.8952 (± 2.474494)	9.26916 (± 0.692768)	2.63062 (± 1.498303)	0.082	< 0.001	< 0.001
**Baseline GSH/GSSG ratio**	9.66667 (± 2.828427)	26.77778 (± 15.435649)	54.90476 (± 13.814531)	0.591	0.007	0.043
**Baseline AP-sites (AP sites/10^5bp)**	49.83333 (± 7.778175)	22 (± 10.969655)	13.04762 (± 4.386125)	0.005	< 0.001	0.267
**NER capacity AUC [(Arbitrary units)x h]**	252.045 (± 127.102444)	48.09667 (± 3.762636)	37.75 (± 20.217226)	0.115	0.047	1.000
**ICL/R capacity AUC [(adducts/10^6nucl)x h]**	12345.5 (± 303.348809)	700.11111 (± 82.786428)	658.28571 (± 264.370561)	0.048	0.062	1.000

Significance values have been adjusted by Bonferroni correction for multiple tests ; Group A: MM1S, OPM2; Group B: LP1, RPMI-8226, SKMM2; Group C: AMO1, NCI-H929, U266, XG-7, XG-6, RPMI-1788; SD: Standard Deviation; AUC: Area under the curve

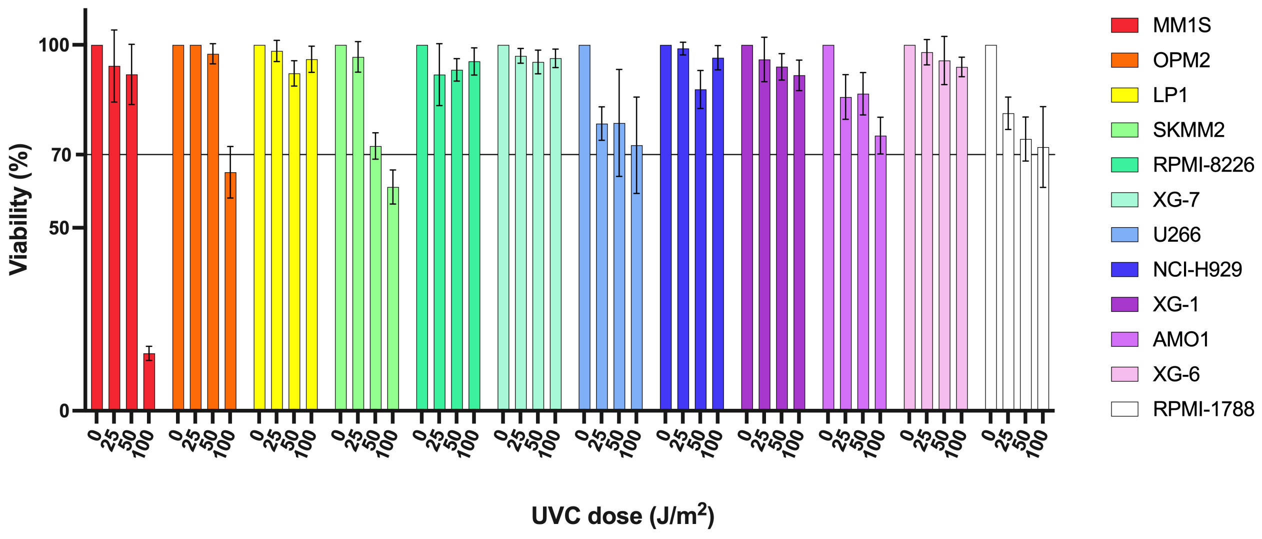

Supplementary Figure S1. UVC-induced sensitivity. Cell viability was assessed by MTT assay 6 hours post treatment. Values were normalized to internal controls and are presented as percentage. Each experiment was performed at least three independent times. Error bars represent SD.

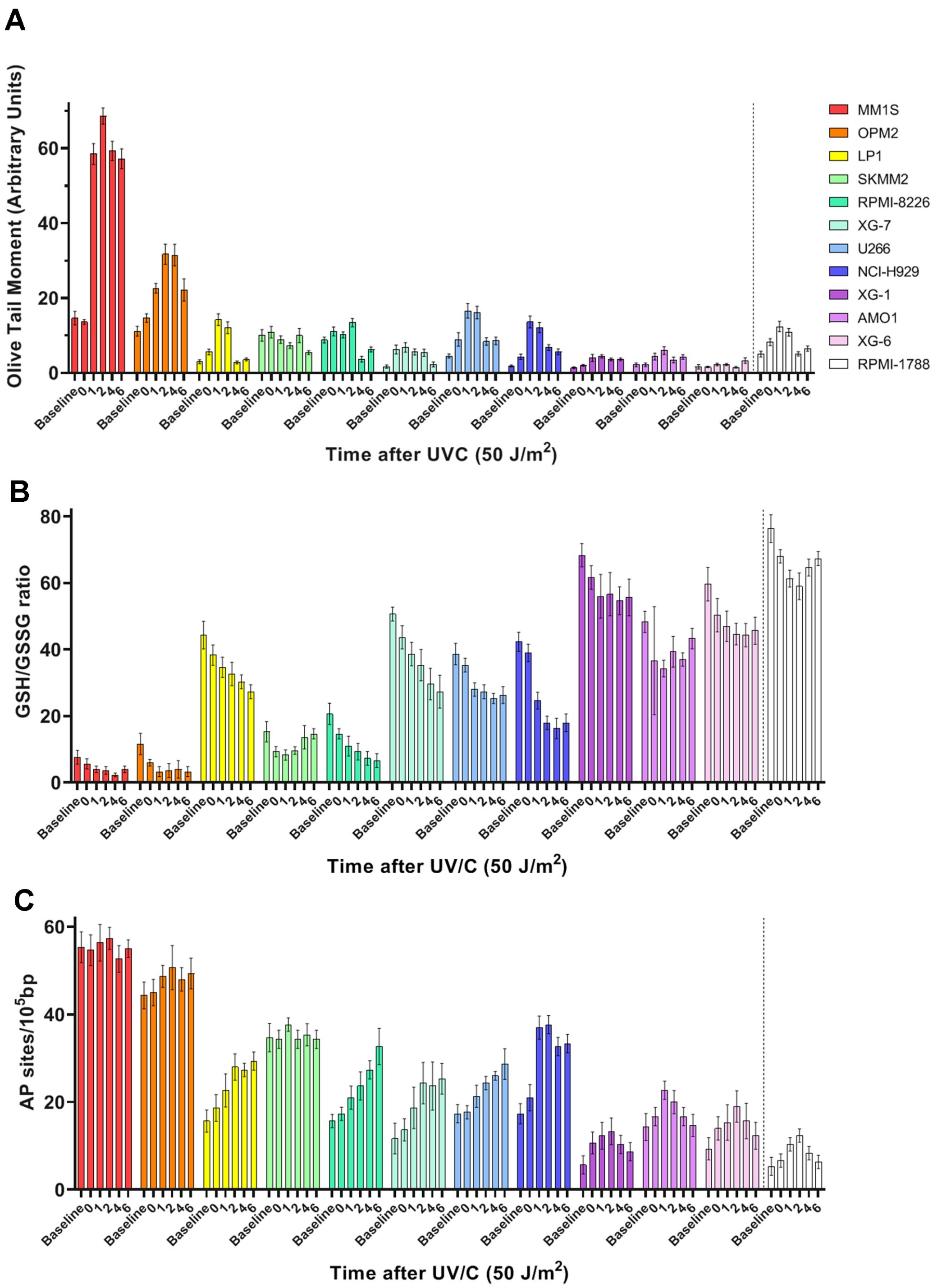

Supplementary Figure S2. UVC-induced DDR signals in MM cell lines. (**A**) Bar graphs showing the kinetics of UVC-induced NER repair using the alkaline comet assay. (**B**) GSH/GSSG ratio, and (**C**) AP-sites kinetics post-UVC irradiation. A minimum of three biological independent replicates was performed for each experiment. Error bars represent SD.

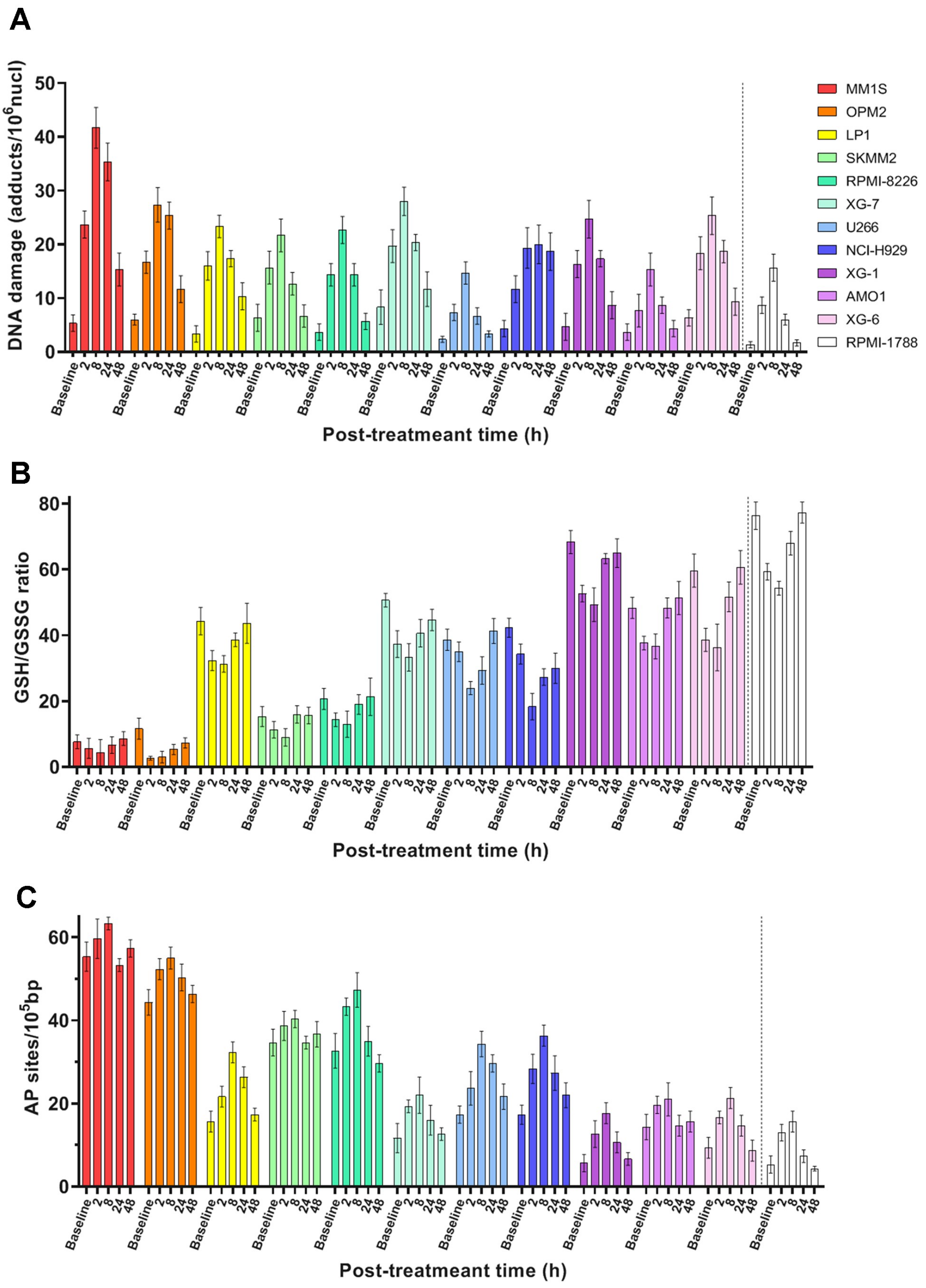

Supplementary Figure S3. DDR-related signals in MM cells following melphalan treatment. (**A**) Bar graphs showing the kinetics of ICLs formation and repair following treatment with 100μg/ml melphalan. (**B**) GSH/GSSG ratio, and (**C**) AP-sites formation at multiple timepoints following treatment. All experiments were conducted with at least three independent replicates. Error bars represent SD.

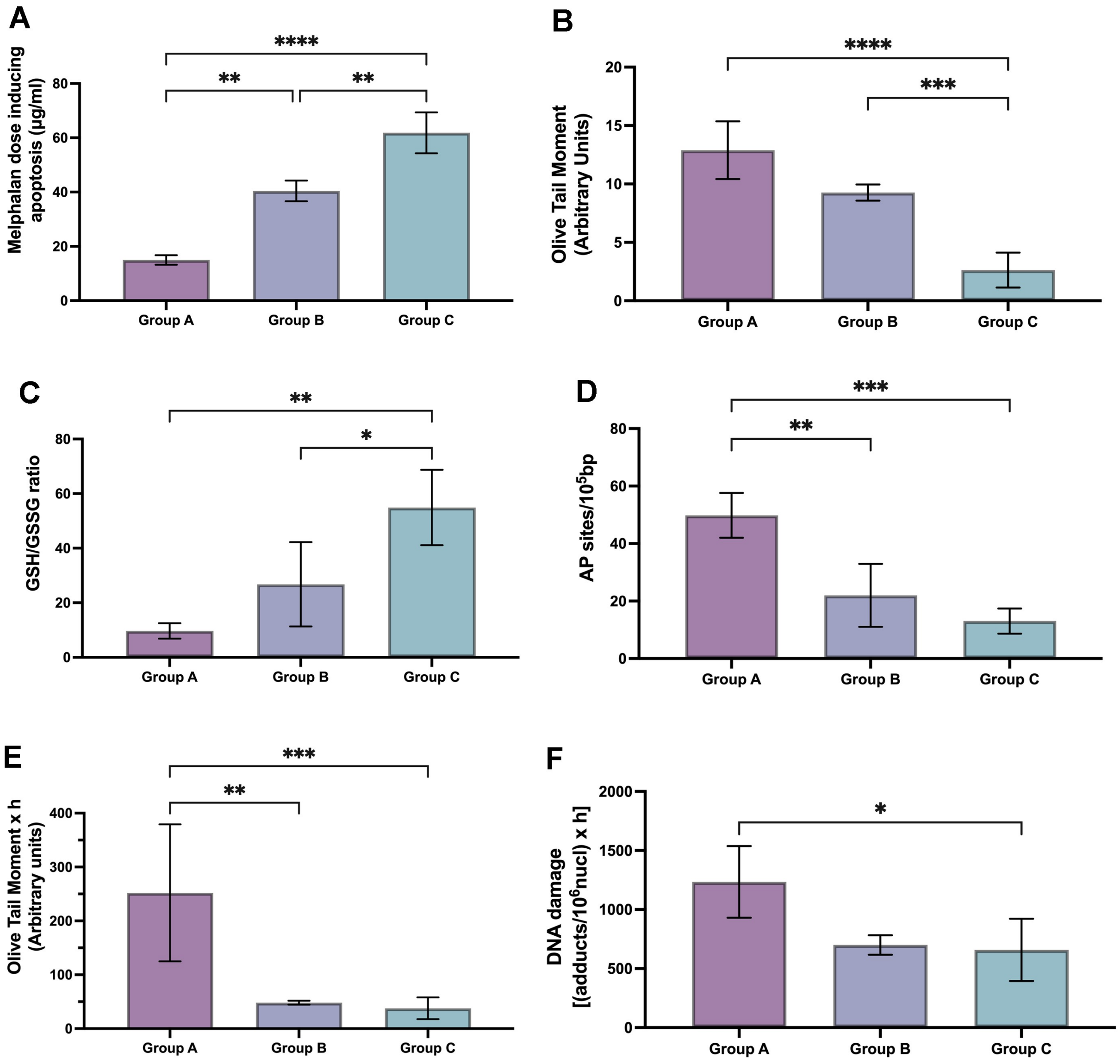

Supplementary Figure S4. One-way ANOVA results on the DDR and redox markers among the three groups generated from clustering analysis. (**A**) Apoptosis rates. (**B**) Endogenous/baseline DNA damage. (**C**) Baseline GSH/GSSG ratio. (**D**) Baseline AP-sites. (**E**) NER capacity (AUC). (**F**) ICL/R capacity (AUC). Bars represent mean±SD. Statistical significance is indicated as follows; *$p \leq 0.05$, ** $p \leq 0.01$, *** $p \leq 0.001$, **** $p \leq 0.0001$.
